# Manual acupuncture versus sham acupuncture and usual care for the prevention of primary dysmenorrhea (PD): study protocol for a randomized controlled trial

**DOI:** 10.1186/s13063-020-04720-5

**Published:** 2020-09-29

**Authors:** Lingling Yu, Shiqin Liu, Cuihong Zheng, Wenhua Liu, Hua Wang, Fengxia Liang, Wei Lu, Shabei Xu, Wei Wang

**Affiliations:** 1 Tongji Hospital, Tongji Medical College, Huazhong University of Science and Technology, 1095 Jiefang Avenue, Wuhan, 430030 Hubei China; 2 Acupuncture and Moxibustion Institution, College of Acupuncture and Moxibustion, Hubei University of Chinese Medicine/Hubei Provincial Collaborative Innovation Center of Preventive Treatment by Acupuncture and Moxibustion, 1 Tanhualin Road, Wuhan, 430030 Hubei China

**Keywords:** Primary dysmenorrhea, Manual acupuncture, Efficacy, Safety, Clinical trial, Study protocol

## Abstract

**Background:**

Primary dysmenorrhea (PD) is a leading cause of dysmenorrhea among adolescent girls. Manual acupuncture may be considered as an effective treatment for PD, but high-quality evidence remains limited. This trial aims to evaluate the efficacy and safety of acupuncture for the prevention of PD as compared with sham acupuncture and usual care.

**Methods/design:**

This is a three-arm, randomized, controlled clinical trial in which the patients, assessors, and statisticians will be blinded. A total of 300 acupuncture-naive patients who were diagnosed as PD will be randomly allocated to the verum acupuncture, sham acupuncture, or usual care groups in a 2:2:1 ratio. Patients in the verum acupuncture group will receive manual acupuncture at specific acupuncture points with penetrating needling, while those in the sham acupuncture group will receive non-penetrating needling at non-acupuncture points. They will be given five sessions over a menstrual cycle for 3 menstrual cycles. Patients in the usual care group will receive health education and informed to receive manual acupuncture for free after waiting for 7 menstrual cycles. The primary outcome will be the change from baseline in the Cox Menstrual Symptom Scale Score (CMSS). The secondary outcomes will be the changes in Massachusetts General Hospital Acupuncture Sensation Scale (MASS), visual analog scale (VAS), Short-Form McGill Pain Questionnaire 2 (SF-MPQ-2), Pittsburgh Sleep Quality Index (PSQI), Beck Anxiety Inventory (BAI), Beck Depression Inventory II (BDI- II), Acupuncture Expectancy Scale (AES), 60-item NEO Personality Inventory-Short Form (NEO-FFI), and acute medication intake. The adverse events will be recorded at every visit. The analyses will be performed base on a full analysis set (FAS) and a per-protocol set (PPS).

**Discussion:**

This study may provide high-quality evidence regarding the efficacy and safety of manual acupuncture for PD. In addition, the results of this study will help to identify the efficacy of acupuncture due to the specific effects of acupuncture or placebo effects of acupuncture ritual.

**Trial registration:**

Clinical Trials.gov NCT02783534. Registered on 26 May 2016

## Background and rationale

Primary dysmenorrhea (PD), defined as painful menstruation in the absence of pelvic pathology, characteristically begins when adolescents attain ovulatory cycles, usually within 6–12 months of menarche [1]. It is estimated to affect 93% of adolescents in Australia [2], 68.1–72.2% in Turkey [3], 56.15% in India [4], 51.1% in China [5], and 46.8% in Japan [6], with a very severe pain accounting for 5.4 to 17.7% [5, 6]. The burden of PD is large; it is one of the leading causes of quality-adjusted life year loss in women [7]. In addition, women with PD had significantly higher scores of depression and anxiety than those without PD [8]. The current medical treatment for PD is symptomatic, often with nonsteroidal anti-inflammatory drugs and combined oral contraceptives pills [9]. However, the failure rate may reach up to 20–25% [9]. Hence, an effective and safe therapy for PD prophylaxis is still required.

Acupuncture is an important component of traditional Chinese medicine and may offer a non-pharmacological approach for PD prophylaxis. Many clinical trials have demonstrated the efficacy of acupuncture in the relief of PD. Such studies have been summarized in several recent reviews [10, 11]. However, the available evidence of the efficacy of acupuncture for PD remains insufficient because many clinical trials have had small sample, short follow-up, risk of bias, or other methodological limitations.

In clinical practice, two acupuncture manipulations were widely used: manual acupuncture and electrical acupuncture. Manual acupuncture is used as an analgesic therapy for more than 2000 years and is usually practiced by traditional acupuncturists. It is defined as the insertion of fine needles into specific acupoints and followed by the manual manipulation (i.e., intermittent rotation as well as lift and thrust) of needles [12]. The analgesic effects of manual acupuncture manifest when the stimulation reached a threshold allowing to generate *de qi* sensations [12]. In animal experiments, acupuncture needle rotation can trigger an increased release of adenosine and endogenous opioids and in turn regulate the analgesic effects of manual acupuncture [13, 14]. Therefore, the current trial is designed to evaluate the effect of manual acupuncture for the prevention of PD.

### Objectives

Through this study, we expect to test the following hypotheses: (1) evaluate whether manual acupuncture is superior to sham acupuncture and usual care for the prevention of PD and (2) identify whether the efficacy of acupuncture is due to the specific effects of acupuncture or placebo effects of acupuncture ritual.

### Trial design

**Fig. 1:**
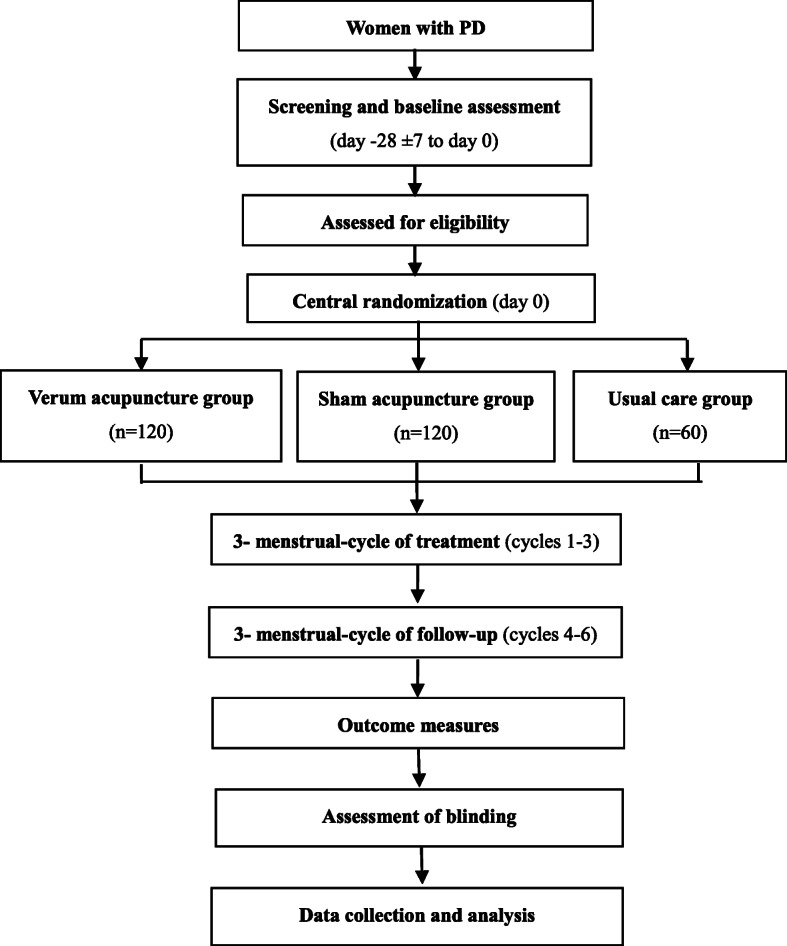
Flowchart of the trial procedures

This will be a multicenter, stratified, three-arm, randomized, controlled trial. A total of 300 eligible patients will be randomized in a 2:2:1 ratio to receive verum acupuncture, sham acupuncture, or usual care. This trial has been registered on the ClinicalTrials.gov protocol registration system (NCT02765581). The study design is presented in the flowchart in Fig. 1. The Standard Protocol Items: Recommendations for Interventional Trials (SPIRIT) checklist is provided as Additional file 1.

## Methods

### Study setting

This study will be conducted in the following five academic hospitals in China: Tongji Hospital, affiliated to Huazhong University of Science and Technology; Hubei Provincial Hospital of Traditional Chinese Medicine; University Hospital of Huang Jia Lake Campus, affiliated to Hubei University of Chinese Medicine; Wuhan Hospital of Integrated TCM and Western Medicine; and TCM Clinic of Hubei University of Chinese Medicine.

### Inclusion criteria

The following are the inclusion criteria:

1. Being diagnosed with PD according to the Primary Dysmenorrhea Consensus Guideline established by the Canadian Institute of Obstetrics and Gynecology in 2005 [15]
2. Aged between 15 and 40 without a history of delivery
3. Have a history of regular menstrual cycles (28 days ± 7 days)
4. Experiencing an average menstrual pain visual analog scale (VAS) score between 40 and 80 for at least 6 months
5. Acupuncture naive
6. Able to complete the baseline PD diary
7. Able to sign an informed consent

### Exclusion criteria

The following are the exclusion criteria:

1. Secondary dysmenorrhea caused by endometriosis, pelvic inflammation, uterine myomas, or other gynecological problems confirmed by type B ultrasound exam by gynecologists
2. Having a history of pelvic or abdominal surgery
3. Combined with uncontrolled diagnosed psychiatric disorders such as severe anxiety and depression, or severe systemic diseases affecting the implementation of treatment programs
4. Received any other treatment for PD 3 months prior to enrollment
5. Pregnant women, women in lactation, and those planning to become pregnant
6. Experience of acupuncture
7. Illiterate or patients unable to read and understand questionnaires

### Consent to participate

This study will be conducted in accordance with the Declaration of Helsinki [16]. After being informed of the potential benefits and risks, all subjects will be required to provide informed consent prior to enrollment in the trial. In each sub-center, two independent gynecologists are responsible for recruiting the patient and gaining informed consent. On the consent form, participants will be asked if they agree to the use of their data if they choose to withdraw from the trial. Participants will also be asked for permission for the research team to share relevant data with people from the universities taking part in the research or from regulatory authorities, where relevant. This trial does not involve collecting biological specimens for storage. No changes are made to the trial protocol after the subject recruitment started.

### Interventions

To improve patients’ adherence to interventions, acupuncture will be performed by acupuncturists with over 5 years of clinical acupuncture experience and obtained a license from the Ministry of Health of the People’s Republic of China. Before performing the treatment, acupuncturists will receive centralized training. The training courses include an introduction to the basic clinical research methods and a practical demonstration of the treatment. Patients will be treated once a day for 5 consecutive days (a treatment course), start from the 5th or 7th day before the estimated first day of the latest menstrual cycle. A total of 3 consecutive treatment courses will be administrated. Each treatment session will be 30 min in duration. Streitberger acupuncture needles and Streitberger placebo needles will be used for the treatment, which is proven to be a validated and reliable single-blind acupuncture needle used to investigate the effects of acupuncture [17].

### Manual acupuncture

For the verum acupuncture group, manual acupuncture treatment will be applied to both obligatory and additional points using Streitberger acupuncture needles. The obligatory points include RN4 and bilateral SP 6, EX-CA1, and SP10. Additional points will be chosen according to different syndromes: bilateral ST36 for the deficient syndrome and bilateral SP8 for the sufficiency syndrome. Table 1 describes the acupuncture point prescription. After sterilizing the skin, sterile and disposable Streitberger acupuncture needles (0.30 mm in diameter and 30 mm in length) will be inserted into the acupuncture points. *De qi* sensations will be obtained through the manipulation of needles (rotating the needles 80–120 times per minute). During the 30-min acupuncture session, the manipulation of needles for each acupuncture point will last 10 s and will be repeated 4 times to induce lasting *de qi* sensations.

**Table 1:** Details of the acupoints used in the verum acupuncture group

Acupoints (standard abbreviation/Chinese nomenclature)	Location	Indication	Insertion depth (cun*)
RN4/Guanyuan	On the anterior midline, 3 cun^a^ below the umbilicus	Coordinates Chong and conception vessels	1 to 1.5
SP6/Sanyinjiao	3 cun directly above the tip of the medial malleolus	Nourishes kidney and liver yin	1 to 1.5
EX-CA1/Zigong	4 cun below the umbilicus and 3 cun lateral to the anterior midline	Regulates the menstrual cycle and relief menstrual pain	0.8 to 1.2
SP10/Xuehai	2 cun above the upper border of the medial patella when the knee is fixed	Activates blood circulation to dissipate blood stasis	1 to 1.5
ST36/Zusanli	One-finger width lateral to the anterior border of the tibia and 3 cun directly below the lateral depression of the patellar ligament	Nourishes the spleen and stomach and coordinates qi and blood	1 to 2
SP8/Diji	On the line joining the tip of the medial malleolus and posteroinferior to the medial condyle of the tibia and 3 cun below the medial condyle of the tibia	Nourishes the spleen and disinhibit uninhibited dampness	1 to 2

*A cun is a measurement used to locate acupoints and corresponds to the distance between the two medial ends of the creases of the interphalangeal joints when the patient’s middle finger is flexed

### Sham acupuncture

For the sham acupuncture group, Streitberger placebo needles will be applied at 510 non-acupuncture points, located bilaterally on the back, at a distance of 5 cun lateral to the sixth, seventh, eighth, ninth, and tenth thoracic spine, respectively. No meridians pass through the location of these non-acupuncture points. Because the Streitberger placebo needle is non-penetrating, the acupuncturist will fix a small plastic ring over the point, and then stick the needle through the plaster inside the ring. This will fix the needles on the surface of these non-acupuncture points. Other acupuncture rituals will be identical to the verum acupuncture group.

### Usual care

For the usual care group, patients will be provided with 15 sessions of manual acupuncture treatment for free after a waiting period of 7 menstrual cycles. All of the three groups will receive health education. Health education will include the following aspects: (1) patients will be encouraged to engage in an appropriate exercise training program, (2) patients will be instructed to avoid raw and cold food, and (3) patients will be given relaxing training and psychological counseling.

### Co-interventions

In order to avoid confounding factors in the results of the trial, patients will be asked to avoid commencing other co-interventions for PD during the whole period of this study. In case of severe pain, diclofenac sodium enteric-coated tablets (25 mg/tablet; maximal tolerated dose = 200 mg/day) will be allowed as a rescue medication. Patients will be instructed not to take any other analgesics.

### Criteria for discontinuing interventions

Acupuncture will be ended if participants meet any of the following conditions:

1. Had serious adverse events during or after treatment
2. Misdiagnosed after randomization
3. Patients found to be pregnant after randomization

### Provisions for post-trial care

There is no anticipated harm and compensation for trial participation.

### Outcome measures

#### Primary outcome measure

##### Cox Menstrual Symptom Scale

In this study, the primary outcome will be the changes in Cox Menstrual Symptom Scale (CMSS) scores from baseline to menstrual cycle 6 after randomization based on the dysmenorrhea diaries. The changes in CMSS from baseline to other menstrual cycles (menstrual cycles 1–5 after randomization) will also be assessed. CMSS has been widely used for integrally evaluating patients’ dysmenorrhea symptoms [18]. In the severity evaluation, each symptom is scored via five levels, 0 denotes that the symptom is not noticeable, while 4 denotes it as very severely bothersome. In the duration evaluation, each symptom is scored in five grades, 0 denotes that the symptom did not occur, while 4 denotes that it lasted several days.

#### Secondary efficacy outcome measures

##### Massachusetts General Hospital Acupuncture Sensation Scale

Massachusetts General Hospital Acupuncture Sensation Scale (MASS) has been proposed as a useful tool for measuring *de qi* sensations in acupuncture research [19]. It includes 12 descriptors (soreness, aching, deep pressure, heaviness, fullness/distension, tingling, numbness, sharp pain, dull pain, warmth, cold, and throbbing) that are associated with the acupuncture treatment and one additional field allowing patients to describe the acupuncture sensation in their own words. Prior to the first treatment, the acupuncturist will give participants a brief description of *de qi* sensations. Participants will be asked to rate the intensity of experienced *de qi* sensations immediately after each acupuncture session. Patients in the usual care arm will not receive this questionnaire.

##### Visual analog scale

VAS is a simple and frequently used method for the assessment of variations in the intensity of pain [20]. We will use a 10-mm (0 = no pain, 10 = worst pain ever) VAS to assess the efficacy of acupuncture for pain relief. VAS score of each menstrual pain day will be recorded in a dysmenorrhea diary. The VAS will be assessed at baseline and during the first, second, third, fourth, fifth, and sixth menstrual cycles after randomization.

##### Short-Form McGill Pain Questionnaire 2

Short-Form McGill Pain Questionnaire 2 (SF-MPQ-2) is a highly reliable instrument to evaluate neuropathic and non-neuropathic pain conditions [21]. It is used as a complementary index of pain relief. The SF-MPQ-2 will be assessed at baseline and during the first, second, third, fourth, fifth, and sixth menstrual cycles after randomization.

##### Pittsburgh Sleep Quality Index

Pittsburgh Sleep Quality Index (PSQI) is a self-rated questionnaire which assesses sleep quality and disturbances over a 1-month time interval [22]. It has 19 questions which can be grouped into seven categories: subjective sleep quality, sleep latency, sleep duration, habitual sleep efficiency, sleep disturbances, use of sleeping medication, and daytime dysfunction. The PSQI will be assessed at baseline and during the first, second, third, fourth, fifth, and sixth menstrual cycles after randomization.

##### Intake of acute medication

The intake of diclofenac sodium enteric-coated tablets will be recorded in the dysmenorrhea diary and will be calculated during each menstrual cycle.

#### Secondary psychological outcome measures

##### Beck Anxiety Inventory

The 21-item Beck Anxiety Inventory (BAI) self-report questionnaire is a reliable and valid measure of anxiety severity [23]. In this study, BAI will be measured at baseline, menstrual cycle 3, and menstrual cycle 6 after randomization.

##### Beck Depression Inventory II

BDI has good sensitivity and specificity for major depression in chronic pain patients [24]. It contains 21-item, cutoff scores of > 20 (high) and < 14 (low) were adopted for depression symptoms. Beck Depression Inventory II (BDI-II) will be measured at baseline, menstrual cycle 3, and menstrual cycle 6 after randomization.

##### NEO Personality Inventory-Short Form

The 60-item NEO Personality Inventory-Short Form (NEO-FFI) is a self-administered personality test, scored on the five domains of neuroticism, extraversion, openness, agreeableness, and conscientiousness [25]. It has been widely used for pain patients and demonstrates good psychometric properties [26]. NEO-FFI was only measured once at baseline.

##### Acupuncture Expectancy Scale

Acupuncture Expectancy Scale (AES) is a simple four-item instrument with a valid and reliable score that measures expectancy about acupuncture therapy and correlates to subject reported response [27]. Patients in the verum acupuncture and sham acupuncture groups will be assessed using AES before the first acupuncture session and after completing the third and fifth acupuncture sessions. Patients in the usual care arm will not receive this questionnaire because they only receive compensatory acupuncture treatment after completing the whole study.

### Adverse events

The frequency of adverse events (AEs) will be recorded during the treatment period. AEs will not be recorded in the usual care arm.

### Assessment of blinding

After patients completed the whole visit, they will be asked to guess which type of acupuncture they received.

### Participant timeline

The participant timeline is described in Table 2.

**Table 2:** Participant timeline

Period	Baseline		Treatment			Follow-up		
Menstrual cycle	− 1	0	1	2	3	4	5	6
**Participants**								
Screening	√							
Demography	√							
Informed consent	√							
PD diary	√		√	√	√	√	√	√
Randomization		√						
**Interventions**								
Verum acupuncture			√	√	√			
Sham acupuncture			√	√	√			
Usual care			√	√	√			
**Outcomes**								
CMSS	√		√	√	√	√	√	√
VAS	√		√	√	√	√	√	√
SF-MPQ-2	√		√	√	√	√	√	√
PSQI	√		√	√	√	√	√	√
BAI	√				√			√
BDI- II	√				√			√
NEO-FFI	√							
AES			√		√			
MASS			√	√	√			
**Trial safety evaluation**								
Adverse events			√	√	√			
Assessment of blinding								√

*AES* Acupuncture Expectancy Scale, *BAI* Beck Anxiety Inventory, *BDI-II* Beck Depression Inventory II, *CMSS* Cox Menstrual Symptom Scale, *MASS* Massachusetts General Hospital Acupuncture Sensation Scale, *NEO-FFI* NEO Personality Inventory-Short Form, *PD* primary dysmenorrhea, *PSQI* Pittsburgh Sleep Quality Index, *SF-MPQ-2* Short-Form McGill Pain Questionnaire 2, *VAS* visual analog scale

### Sample size

The sample size was estimated using the Statistical Analysis System software program (version 9.3; SAS Institute Inc., Cary, NC, USA). According to our pilot trial, we anticipate a greater CMSS score decline of 0.2 ± 0.55 in the verum acupuncture group compared with the sham acupuncture group. This sample size calculation is based on a superiority design with a power level of 80% and a significance level set at 5%, with a 1:1 ratio. Based on these assumptions, 170 patients will be needed, with 85 patients in each group. Moreover, we anticipate a greater CMSS score decline of 0.5 ± 1.15 in the verum acupuncture group compared with the usual care group. This sample size calculation is based on a superiority design with a power level of 80% and a significance level set at 5%, with a 2:1 ratio. Based on these assumptions, 150 patients will be required, including 100 patients in the verum acupuncture group and 50 patients in the usual care group. This sample size will be inflated to 300 to account for an attrition rate of 20%. Thus, we plan to enroll 300 patients, including 120 patients in the VA and SA groups and 60 patients in the usual care group.

### Recruitment

Eligible patients will be recruited through three strategies. First, patients will be recruited from the outpatient and inpatient clinics of the five hospitals. Second, patients will be recruited through Public Accounts of hospitals. Third, patients will be recruited from nearby communities and schools through the printed recruitment posters.

### Allocation

After completing a baseline evaluation, eligible participants will be randomly allocated to the verum acupuncture group, the sham acupuncture group, or the usual care group in a 2:2:1 ratio by an independent investigator. The 2:2:1 ratio was used to facilitate recruitment and increase the compliance of patients. The randomization sequences will be generated in a centralized manner via an interactive web response system with stratified centers and block size of 5. The independent investigator will provide the random sequence and group assignment immediately to the project acupuncturist within opaque, sealed envelopes. To prevent selection bias, the acupuncturists will be the only persons to know the patient’s group assignment prior to treatment. Central randomization has strict limits regarding authority; no one can check the files except the top principal investigator.

### Blinding

In this study, many efforts will be done to maintain blinding in participants assigned to the verum acupuncture and sham acupuncture groups. For example, eligible patients will be acupuncture naive, we will set up the standardized ritual operations in the two groups as far as possible. However, patients in the usual care group will not be blinded. They will be provided with 15 sessions of manual acupuncture treatment for free after a waiting period of 7 menstrual cycles. This will increase the compliance of patients allocated to the usual care group. In addition, the acupuncturists will not be blinded.

We will try to ensure that the outcome assessors and statisticians are unaware of the group allocation by (1) using A, B, and C to label the three groups, (2) will randomly assign outcome assessors to follow-up, (3) allocation sequence will be concealed until the end of the study. In addition, we will separately give relevant data of MASS, AEs, and AES to an independent statistician for analysis, while the statistician responsible for the analysis of primary outcome and other secondary outcomes will be blinded to group allocation.

### Data collection and management

Demographic and epidemiological data will be obtained at visit 1, using a general information questionnaire. The questionnaire includes information on age, gender, education, occupation, weight, height, previous disease history, history of PD, and menstrual cycle. If the participants are assessed eligible, they will be asked to record dysmenorrhea diary from menstrual cycle 1 before randomization to menstrual cycle 6 after randomization. All of the primary outcome measures and most of the secondary outcome measures will be obtained from the dysmenorrhea diary. The dysmenorrhea diary includes questionnaires on CMSS, VAS, SF-MPQ-2, PSQI, usage of acute medication, BAI, BDI- II, and NEO-FFI. The diary will be separately collected from the participating hospitals every menstrual cycle. Data of AEs and MASS will be recorded on acupuncture report forms by patients after each treatment. The data will be collected up to the time of withdrawal and will be included in the analysis unless participants specifically request for their data to be withdrawn.

Data management will be ensured by an electronic data capture (EDC) system hosted at the Data Management Center of Tongji Hospital. All paper-based data will be checked and input into the EDC system by two research assistants using a separate account and password. After that, paper-based data will be stored in a locked file cabinet in the principal investigator’s office at the Department of Neurology of Tongji Hospital. The electronic data will be stored at the EDC system after being approved by an electronic signature. After approval of a proposal and with a signed data access agreement, researchers whose proposed use of the data has been approved can access the data. Independent randomization, rigorous blinding, and data management make sure the confidentiality of this trial.

### Statistical methods

Data analysis will be performed by an independent statistician using SAS statistical software (version 9.4; SAS Institute Inc.). Wilcoxon rank-sum tests will be used to perform between-group comparisons regarding the CMSS scores (primary outcome). The analysis of variance (ANOVA) will be used to test the difference between the two intervention groups with the baseline date as a variate and taking into account the multicenter effect. The intensity ratings of each individual sensation, the overall sum of all sensations, and the MASS index across treatment modalities were analyzed using a one-way ANOVA and post hoc *t* tests (푃 < .05). Regarding the proportions and the multicenter effect, the chi-square test or the Fisher exact probability test will be used. The CMH-x^2^ test will be used for a drop-out rate comparison, the categorical variable, and compliance. The latter is considered good only when comprised between 80 and 120%. A *p* value < 0.05 will indicate statistical significance (two-sided). In the descriptive statistics analysis, the mean and standard deviation will be used for continuous indexes with normal distribution, and medium and interquartile range will be used for continuous indexes with non-normal distribution. Categorical indicators will be described as case number and frequency.

These analyses will be performed on the base of a full analysis set (FAS) and a per-protocol set (PPS). According to the intention-to-treat analysis strategy, any participant who passes the randomized stage and who receives at least one intervention will be analyzed using a FAS with missing data for the important outcomes interpolated with the last observation carried forward. The PPS will be considered as a supportive analysis. The results of FAS and PPS analyses will be compared to ensure that the results are consistent.

### Monitoring and quality control

To guarantee the quality of the study, unified training before subject recruitments will ensure the researcher’s adherence to the study protocol. Strict eligible criteria limit the study population to a specific scope and reduce the differences among them. A web-based central randomization system is adopted to avoid selection bias. Independent outcome assessment and statistics are designed to guarantee the objectivity of the data.

Quality control of the data will be handled at three different levels. The first level is the quality check of sub-centers regularly by the head of the sub-center. The second level is the quality inspection by the Clinical Research Center of Tongji Hospital, at least once per 3 months. The third level is the quality monitor by the Clinical Evaluation Center of Hubei University of Chinese Medicine, at least once per 6 months.

Treatment safety is based on the assessment of AEs and serious adverse events (SAEs). An event can be defined as an adverse event if subjects suffer a symptom related to acupuncture, which includes unbearable acupuncture pain, palpitation, fainting, dizziness, sweating, needle broking, needle body wound muscle fiber, bleeding at the acupuncture site, and local hematoma. All subjects will be informed of the potential AEs before signing the informed consent. If any AEs occur during the study periods, they will be recorded in detail on the CRF. The researcher should record the following details: the date, the time, the symptoms, and the degree of occurrence related to the treatment at each visit, and provide proper medical advice and care. In order to ensure the safety of subjects, a Data and Safety Monitoring Board (DSMB) was set up to monitor the performance and safety of the trial, which is composed of 5 experts from different fields on the Chinese mainland. If any serious adverse events occur, they will be immediately reported to the DSMB, although SAEs will not be anticipated.

## Discussion

Manual acupuncture is a non-pharmacological treatment that is extremely effective for pain relief and usually performed by traditional Chinese acupuncturists in clinics. Since acupuncture was proposed as a therapeutic intervention for 43 pain conditions by the NIH consensus, the analgesic effect of acupuncture has become more accepted in the Western countries. The underlying mechanisms on acupuncture analgesia maybe involving the release of endogenous opioids, cannabinoids, and adenosine at the central and peripheral systems [12–14].

Recently, a large body of evidence supports the efficacy and safety of acupuncture for the management of PD [28, 29]. This evidence gave rise to a global effort to develop a new generation of treatment options for patients with PD. But so far, high-quality clinical evidence for the role of acupuncture in PD is still insufficient because many clinical trials have had small sample sizes, short follow-up, or other methodological limitations. This study is designed to clarify the efficacy and safety of manual acupuncture for the prevention of PD and to identify the efficacy of acupuncture due to the specific effects of acupuncture or placebo effects of acupuncture ritual. This trial has the strengths of large sample sizes and extended follow-up. The design of the study also meets the methodological benchmarks with valid control, adequate randomization and allocation concealment procedures, and blinding of outcome assessors and statisticians.

The form of acupuncture used in this trial is manual acupuncture treatment, which will be performed by licensed traditional Chinese acupuncturists. After the acupuncture needle is inserted into the acupoints, the manual manipulation of the needle will generate *de qi* sensations and enhance the clinical benefit of acupuncture analgesia. This may be due to the activation of the pain-inhibiting system in the spinal cord and diffuse noxious inhibitory controls (DNIC) [30, 31]. The intensity of *de qi* sensation is critical for the effects of acupuncture [32]. Our earlier study showed that among patients with Bell palsy, acupuncture with strong stimulation that elicited *de qi* had a greater therapeutic effect, and stronger intensity of *de qi* was associated with better therapeutic effects [33]. However, the important concept of *de qi* sensations has been neglected in acupuncture research in Western countries. This may be due to the difficulty to quantitatively measure complex *de qi* sensations in acupuncture research. In this study, we will use the MASS to quantify the intensity of *de qi* sensations after each treatment. Using the MASS, we will explore the association between the intensity of *de qi* sensations and changes in efficacy outcomes. As far as we know, this is the first acupuncture trial to quantify the measure *de qi* sensations in a patient population of PD.

Appropriate control is one of the largest methodological challenges in acupuncture research. So far, most of the clinical trials used penetrating sham acupuncture to control the specific effect of acupuncture, including superficial needling, needling non-specific acupoints, or needling non-acupoints. However, it is increasingly regarded that penetrating sham acupuncture is not a proper control method because it was not totally inert [34]. An advantage of this trial is the use of non-penetrating sham acupuncture with the Streitberger placebo needle, which could mimic an acupuncture procedure without penetrating the skin. It is designed for subject blinding and sufficiently credible to investigate the effects of acupuncture [35], since the spinal segmental relationship between pain sites and acupuncture points partially underlies the functional specificity of acupoints. Consequently, we apply sham acupuncture at ten non-acupuncture points on the back which is beyond the uterus segment to highlight the functional specificity of acupoints in the verum acupuncture treatment group.

Many psychological factors are regarded as risk factors of dysmenorrhea and should be considered in the management of dysmenorrhea [36, 37]. A study indicated the important impact of depression and anxiety on menstrual symptoms among non-smokers patients [38]. In addition, the placebo effects of acupuncture observed in sham-controlled clinical trials may be referred to the psychological benefits of treatment, including acupuncture ritual, expectation, and explanation [26, 39], while depression, anxiety, and personality pathology were considered to have negative impacts on the physiological response to acupuncture [39]. For these reasons, we will comprehensively assess the influence of depression, anxiety, expectation for acupuncture, and patient-doctor relationship in this study.

One limitation of this study is that acupuncturists who will deliver the treatment will not be blinded to the intervention. To reduce their effect on patients, acupuncturists will only know the patient’s group assignment just before treatment. Through this rigid research, we anticipate to provide high-quality evidence on the efficacy of acupuncture for the prevention of PD. If effective, it will offer an important treatment option for patients with PD.

## Trial status

At the time of submission of this protocol (version number: 1.1, version date: Jan 22, 2016), participant enrolment is progressing well and data collection is well underway. Participant recruitment started on August 2, 2016, and is expected to end on July 1, 2021.

## Supplementary information

**Additional file 1.** SPIRIT 2013 Checklist: Recommended items to address in a clinical trial protocol and related documents.

## Data Availability

Not applicable

## Abbreviations

AES: Acupuncture Expectancy Scale
AEs: Adverse events
ANOVA: Analysis of variance
BAI: Beck Anxiety Inventory
BDI-II: Beck Depression Inventory II
CMSS: Cox Menstrual Symptom Scale
DNIC: Diffuse noxious inhibitory controls
EDC: Electronic data capture
FAS: Full analysis set
MASS: Massachusetts General Hospital Acupuncture Sensation Scale
NEO-FFI: NEO Personality Inventory-Short Form
PD: Primary dysmenorrhea
PPS: Per-protocol set
PSQI: Pittsburgh Sleep Quality Index
SF-MPQ-2: Short-Form McGill Pain Questionnaire 2
VAS: Visual analog scale
